# Evaluation of APACHE II, SAPS II and sofa as predictors of mortality in patients over 80 years admitted to ICU

**DOI:** 10.1186/2197-425X-3-S1-A343

**Published:** 2015-10-01

**Authors:** JE Romo Gonzales, J Silva Obregón, C Martin Dal Gesso, P Gallardo Culebradas, S Saboya Sanchez, M Torralba

**Affiliations:** Intensive Care Unit, Hospital Universitario de Guadalajara, Guadalajara, Spain; Anaesthesiology, Hospital Universitario de Guadalajara, Guadalajara, Spain; Intensive Care Unit, Hospital Universitario Puerta de Hierro Majadahonda, Madrid, Spain; Internal Medicine, Hospital Universitario de Guadalajara, Guadalajara, Spain

## Objectives

Evaluate and compare the predictive ability of APACHE II, SAPS II and SOFA in mortality of elderly patients admitted for medical causes in the ICU of the Hospital Universitario de Guadalajara.

## Methods

Retrospective cohort study. In this study we included patients with at least 80 years admitted to ICU for medical cause, from January-2003 to May 2011. Patients with Ischemic heart disease and arrhythmias were excluded. Logistic regressions analysis was used to calculate the sensitivity, specificity and accuracy as well as the OR probability of mortality. The ability to predict mortality was performed with ROC curves.

## Results

There were analyzed 95 patients, 55/95 (58%) males. Median age: 81 years (IQR: 80-83 years). Hospital mortality 52/95 (54.7%). Median APACHE II, SAPS II and SOFA: 23 (IQR 18-29), 7 (IQR: 4-10), 49 (IQR: 40-65).

The sensitivity, specificity and accuracy were respectively 75%, 63% and 69% for APACHE II; 73.1%, 62.8% and 68.4% for SOFA; 73.1%, 76.7% and 74.7% for SAPS II. For every one-point increase in score of APACHE II, SOFA and SAPS II, the possibility of dead increased by 16%, 32% and 13% respectively (p < 0.0001 in all three regressions); coefficient of determination Nagelkerke 0.26, 0.24 and 0.51 respectively. The area under the curve (AUC) of these models was: 0.76 for APACHE II, 0.75 for SOFA and 0.86 for SAPS; the differences between them were statistically significant (p = 0.018).

## Conclusions

In our series, the SAPS II is the model that best predicts mortality in patients with at least 80 years admitted to ICU for medical reasons.

**Figure 1:**
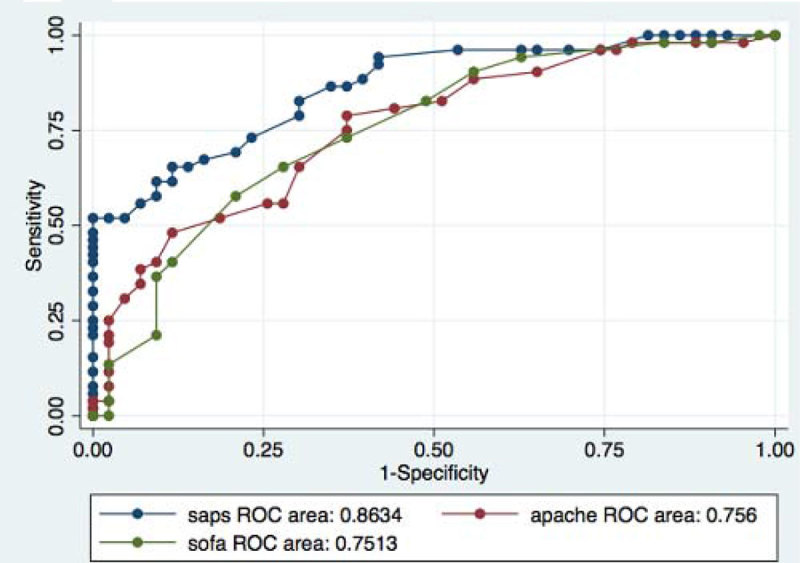
**ROC curve. AUC.**

